# Ruptured Median Raphe Cyst Mimicking a Vascular Penile Mass on Ultrasound

**DOI:** 10.1155/2022/8899541

**Published:** 2022-02-28

**Authors:** Amir Pakray, Andrew Surro, Donald Gibson, Ahmad Tahawi

**Affiliations:** ^1^Department of Diagnostic Radiology, Beaumont Hospital, Royal Oak, MI, Michigan, USA; ^2^Michigan State University College of Osteopathic Medicine, East Lansing, MI, Michigan, USA

## Abstract

Median raphe cysts are uncommon benign cysts thought to occur due to improper fusion of the genital tubercle and can occur anywhere along the median raphe, from the glans to the anus, most commonly occurring along the ventral penile shaft. Limited information is available in the literature about the common imaging features of median raphe cysts with available reports highlighting an avascular cystic lesion. Our case demonstrates a 10-year-old male patient presenting with a ventral penile mass that demonstrated interval growth in the absence of trauma without overlying skin changes. Doppler ultrasound examination demonstrated a solid vascular mass measuring up to 1.6 cm at the ventral aspect of the penis with arterial and venous waveforms. The patient underwent elective resection of the mass which revealed a 2.0 cm inflamed glandular subtype median raphe cyst. This report demonstrates an atypical imaging presentation of an inflamed median raphe cyst, particularly that of a heterogeneous solid mass with arterial and venous blood flow on ultrasound.

## 1. Introduction

Median raphe cysts (MRC) are rare benign cysts known to occur along the median raphe, which extends from the urethral meatus and continues caudally along the scrotum down to the perineum. They are thought to arise due to abnormal fusion of the genital tubercle during the fourth week of fetal development; however, various theories exist in the literature with the exact pathogenesis uncertain [1]. Approximately 100 cases of median raphe cysts have been reported to date. A review of these reports demonstrates encounters by pediatricians, urologists, pathologists, dermatologists, and radiologists, elucidating the importance in understanding the multidisciplinary presentation of MRCs for proper diagnosis and management. A bimodal pattern of presentation has been noted (1-10 years age and 21-40 years age) with the patient typically presenting with cosmetic concerns. [2]. A majority of these lesions are asymptomatic, and when symptomatic, issues with urination and coitus are the most common presenting concerns [2]. Literature has shown that more distal MRCs along the median raphe are more commonly associated with symptoms such as difficulty micturating [2].

MRCs are often misdiagnosed as more common entities such as Cowper gland cysts, dermoid cysts, steatocystomas, or neoplasms depending on the location [3]. The diagnosis of an MRC is typically a clinical one however imaging can be useful to evaluate composition, vascularity, and the extent of the lesion. A definitive diagnosis is ultimately made on histological examination. Various histological subtypes have been demonstrated, most commonly the urethral type which is composed of a urothelium-like epithelium, whereas the glandular subtype occurs less commonly [2, 3]. In fact, as noted by Syed et al., a compilation of reviews displayed only 3 glandular subtypes among 55 patients, highlighting the novelty of our case [3]. Ultrasound and MRI allow for anatomical assessment in order to understand the extent of the lesion and to rule out communication with neighboring structures or vascularity. Typical imaging features described in the literature are that of an avascular cyst on ultrasound [3–6]. Similarly, a cystic structure with high T2 signal can be seen on MRI imaging [4, 7]. A urethrogram may be obtained if suspected fistulous urethral communication is not definitively ruled out on US or MRI. Standard of care for MRC treatment involves local excision with primary closure, however, there are case reports noting success with various options from simple aspiration to wide local excision with deroofing for deeper/larger lesions [8]. Delayed intervention can result in inflammation of the MRC increasing the likelihood of iatrogenic urethral injury in addition to the cosmetic and symptomatic manifestations of treatment delay [5].

## 2. Case Presentation

A 10-year-old male with no significant past medical history presented to the emergency department with isolated penile shaft swelling and minimal pain without infectious symptoms, difficulty urinating or hematuria. The patient denied any trauma to the area of concern and was discharged with instructions to follow up with his primary care physician (PCP). An assessment by the PCP on the next day showed the swelling had decreased, and the patient was sent home with hydroxyzine for pruritus and irritation. At the one week follow up with the PCP, the patient displayed continued decrease in edema with a firm palpable mass at the distal ventral aspect of the penis. The patient also reluctantly confirmed that the mass had been present for as long as he could remember, however, he added that it had recently enlarged.

**Figure 1:**
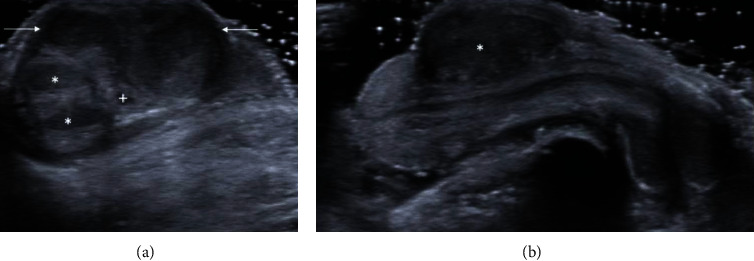
Grey scale ultrasound of the penile median raphe cyst. (a) Transverse view of the distal penile shaft demonstrating the heterogeneous solid mass (arrows) in relation to the corpora cavernosa (∗) and corpus spongiosum (+). (b) Sagittal view demonstrating the solid-appearing median raphe cyst (∗) at the ventrolateral aspect of the distal penile shaft.

**Figure 2:**
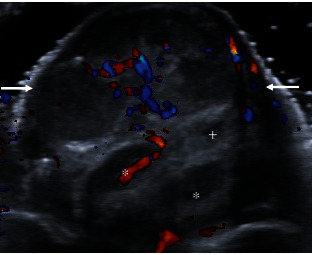
Grey scale ultrasound with Doppler demonstrating Doppler flow to the median raphe cyst (arrows). The corpora cavernosa (∗) and corpus spongiosum (+) are seen distinct from the cyst.

**Figure 3:**
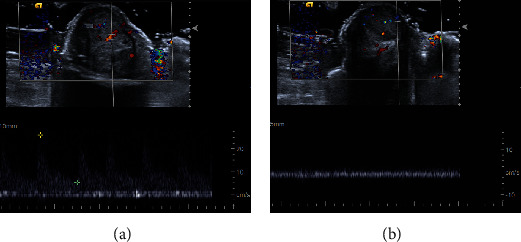
Grey scale ultrasound with spectral waveform analysis. (a) Transverse view demonstrates arterial waveforms within the median raphe cyst. (b) Transverse view showing low resistance venous flow within the median raphe cyst.

Penile ultrasound was then performed which exhibited a heterogeneous solid mass measuring 1.4 × 1.0 × 1.6 cm arising from the subcutaneous tissue of the distal ventral shaft and appeared separate from the left corpus cavernosum and urethra (Figure 1). Color Doppler revealed vascular flow within the lesion (Figure 2), and Spectral Doppler showed arterial and venous waveforms within the lesion (Figure 3). These imaging features are not typical of median raphe cysts or other common cystic lesions, and a neoplastic etiology was favored rather than a benign cystic lesion.

**Figure 4:**
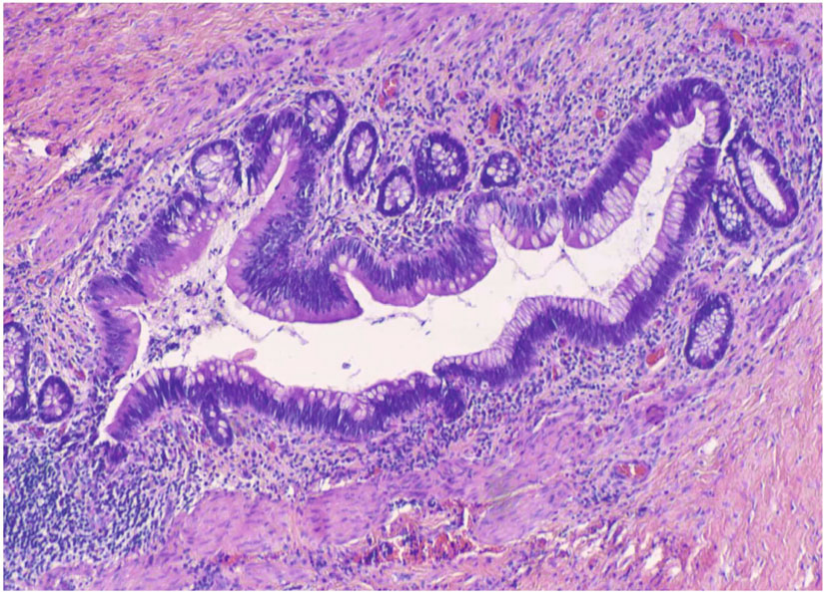
Hematoxylin and eosin stain (original magnification 10×) showing cystic walls lined by mucinous glandular epithelium (glandular type median raphe cyst).

Five days later, the patient was taken to the operating room where a 2 cm mass was identified. The mass was very adherent to the spongiosum and right corporal body on the ventral aspect below the coronal sulcus. The mass appeared cystic in nature and when entered, thick brown/yellow material was encountered. Cultures displayed no growth however many polymorphonuclear leukocytes and red blood cells were seen. Frozen sections demonstrated a benign cystic lesion lined with mucin-producing cuboidal epithelium and urothelium consistent with an inflamed glandular type median raphe cyst with focal rupture (Figure 4). Subsequent cystoscopy confirmed no fistulous tract between the mass and the urethra.

## 3. Discussion

Our case is a novel presentation of a median raphe cyst masquerading as a large vascular penile shaft mass. Typical imaging features of MRCs in the literature are those of an avascular cystic lesion on ultrasound [3–6]. Our case demonstrates several atypical imaging features on ultrasound including an internal echogenicity suggestive of a solid mass. The most atypical feature was internal Doppler flow with arterial and venous waveforms on spectral analysis, which is most consistent with a vascular mass. These imaging features warranted the diagnostic consideration of a neoplastic etiology. Although a penile neoplasm and benign cystic lesion would both be managed operatively, a preliminary diagnosis of a penile neoplasm may cause unnecessary stress to the patient and family. Two case reports, Yu et al. and Parnham et al., describe a solid-appearing lesion on ultrasound, however, both studies demonstrated an avascular mass unlike our case [4, 7]. In the case described by Yu and Capolicchio, the lesion demonstrated a high T2 signal on MRI consistent with a cyst [7]. The median raphe cyst appearing as a soft tissue lesion by Parnham et al. also did not demonstrate vascularity or enhancement on contrast-enhanced imaging [4]. Our case highlights the importance of considering a complicated median raphe cyst in the differential of a midline penile lesion which appears solid and demonstrates internal vascularity.

Given the lack of infectious symptoms or overlying signs of cellulitis, consideration of an infected MRC was not immediately entertained. When an MRC is infected, the patient may present with pain, overlying erythema, or exuding pus typically occurring post coitum [3]. In addition to considering an infected MRC when the history suggests interval increase in size, one must consider rupture as well. In our case, the history of recent enlargement without overt infectious symptoms or history of intercourse may have suggested rupture rather than infection. The patient had no operative complications, and satisfactory results on follow-up were noted. Postoperative fistulous complications have been reported [2], therefore, caution should be taken in cases where inflammation or adhesions are encountered in infected or ruptured MRCs.

Our case also demonstrates the limitations of lesion characterization on ultrasound. Typical cystic penile lesions are seen on ultrasound as avascular anechoic structures and may have internal echoes if complicated by debris such as protein [9]. Acoustic through transmission is another common identifying feature of cysts. Additionally, internal Doppler flow should not be present in cysts whereas it is a feature seen with vascular masses.

## 4. Conclusion

We present a novel case of a ruptured glandular subtype median raphe cyst in a 10-year-old with recent enlargement prior to presentation. A ruptured MRC should be considered in the differential when a solid vascular subcutaneous mass is noted along the median raphe without communication with adjacent structures and lack of clinical infectious findings. A ruptured MRC may present as a relatively large lesion (>1.5 cm) with arterial and venous flow as demonstrated in our case.

## Data Availability

Additional data such as full DICOM images are available from the corresponding author upon request.

## References

[1] Lezcano C., Chaux A., Velazquez E. F., Cubilla A. L. (2015). Clinicopathological features and histogenesis of penile cysts. *Seminars in Diagnostic Pathology*.

[2] Shao I. H., Chen T. D., Shao H. T., Chen H. W. (2012). Male median raphe cysts: serial retrospective analysis and histopathological classification. *Diagnostic Pathology*.

[3] Syed M. M. A., Amatya B., Sitaula S. (2019). Median raphe cyst of the penis: a case report and review of the literature. *Journal of Medical Case Reports*.

[4] Parnham A. S., Freeman A., Kirkham A., Muneer A. (2015). An unusual swelling in the male perineum. *Case Reports*.

[5] Patoulias D., Kalogirou M., Chatzopoulos K., Patoulias I. (2017). Canaliform median raphe cysts (MRCs) lined by squamous epithelium in a 5 year old male patient; report of a rare case and comprehensive review of the literature. *Folia Medica Cracoviensia*.

[6] Hajar C., Hajjali I. R., Oscar L., Mayes D. C. (2019). Median raphe cyst: a clinically challenging diagnosis. *Clinics and Practice*.

[7] Yu A., Capolicchio J. P. (2017). A case of epidermoid median raphe cyst traversing the corpora cavernosa. *Canadian Urological Association Journal*.

[8] Tarate D. C., Tambe S. A., Nayak C. S. (2018). Median raphe cyst of penis. *Indian Journal of Dermatology, Venereology and Leprology*.

[9] Rocher L., Glas L., Cluzel G., Ifergan J., Bellin M. F. (2012). Imaging tumours of the penis. *Diagnostic and Interventional Imaging*.

